# Performance of random forest when SNPs are in linkage disequilibrium

**DOI:** 10.1186/1471-2105-10-78

**Published:** 2009-03-05

**Authors:** Yan A Meng, Yi Yu, L Adrienne Cupples, Lindsay A Farrer, Kathryn L Lunetta

**Affiliations:** 1 Genetics Program, Department of Medicine, School of Medicine, Boston University, Boston, MA, USA; 2 Department of Biostatistics, School of Public Health, Boston University, Boston, MA, USA; 3 Center for Human Genetic Research, Massachusetts General Hospital, Boston, MA, USA; 4 Broad Institute of Harvard and Massachusetts Institute of Technology, Cambridge, MA, USA

## Abstract

**Background:**

Single nucleotide polymorphisms (SNPs) may be correlated due to linkage disequilibrium (LD). Association studies look for both direct and indirect associations with disease loci. In a Random Forest (RF) analysis, correlation between a true risk SNP and SNPs in LD may lead to diminished variable importance for the true risk SNP. One approach to address this problem is to select SNPs in linkage equilibrium (LE) for analysis. Here, we explore alternative methods for dealing with SNPs in LD: change the tree-building algorithm by building each tree in an RF only with SNPs in LE, modify the importance measure (IM), and use haplotypes instead of SNPs to build a RF.

**Results:**

We evaluated the performance of our alternative methods by simulation of a spectrum of complex genetics models. When a haplotype rather than an individual SNP is the risk factor, we find that the original Random Forest method performed on SNPs provides good performance. When individual, genotyped SNPs are the risk factors, we find that the stronger the genetic effect, the stronger the effect LD has on the performance of the original RF. A revised importance measure used with the original RF is relatively robust to LD among SNPs; this revised importance measure used with the revised RF is sometimes inflated. Overall, we find that the revised importance measure used with the original RF is the best choice when the genetic model and the number of SNPs in LD with risk SNPs are unknown. For the haplotype-based method, under a multiplicative heterogeneity model, we observed a decrease in the performance of RF with increasing LD among the SNPs in the haplotype.

**Conclusion:**

Our results suggest that by strategically revising the Random Forest method tree-building or importance measure calculation, power can increase when LD exists between SNPs. We conclude that the revised Random Forest method performed on SNPs offers an advantage of not requiring genotype phase, making it a viable tool for use in the context of thousands of SNPs, such as candidate gene studies and follow-up of top candidates from genome wide association studies.

## Background

Association studies for complex phenotypes consider genotypes for thousands of single nucleotide polymorphisms (SNPs), either derived from genome wide association studies, or candidate gene studies. One approach to dealing with large numbers of SNPs is to screen the data using some criterion to rank SNPs for follow-up. Machine learning approaches can be efficient at selecting from large numbers of predictor variables. In this paper, we evaluate the performance of Random Forests [1], one machine-learning method, in association studies. Previously, Lunetta *et al*. [2] showed that when unknown interactions among SNPs exist in a data set consisting of thousands of SNPs, random forest (RF) analysis can be substantially more efficient than standard univariate screening methods in ranking the true disease-associated SNPs from among large numbers of unassociated SNPs.

Random Forests are built using Classification and Regression Tree methods, Ensemble methods, Bagging, and Boosting with desirable characteristics such as good accuracy; robustness to outliers and noise; speed; internal estimation of error, strength, correlation and variable importance; simplicity and ease of parallelization [1,3-6]. The approach grows many classification trees or regression trees, called "forests", with no trimming or pruning of the fully grown trees. Two stochastic features distinguish Random Forests from deterministic methods. First, every tree is built using a bootstrap sample of the observations. Second, at each node, a random subset of all predictors (the size of which is referred to as *mtry *in this paper) is chosen to determine the best split rather than the full set. Therefore, all trees in a forest are different. For each tree, approximately one third of all the observations are left out of the bootstrap sample; these observations are called "out-of-bag" (OOB) data. The OOB data are then used to estimate prediction accuracy. For a particular tree, each OOB observation is given an outcome prediction. The overall prediction of each individual is then obtained by counting the predictions over all trees for which the individual was out-of-bag, and the outcome with the most predictions is the individual's predicted outcome. This Random Forest method also produces for each variable a measure of importance that quantifies the relative contribution of that variable to the prediction accuracy. The importance score is calculated by randomly permuting the variable's values among the OOB observations for each tree and measuring the prediction error (PE) increase resulting from it and averaging over the total number of trees. This shuffling increases PE if the variable is of high importance and is not affected otherwise. We use this score to prioritize the variables by ranking them.

For any analysis procedure, the more highly correlated the variables are, the more they can serve as surrogates for each other, weakening the evidence for association for any single correlated variable to the outcome if all are included in the same model. Strobl *et al*. (2008) [7] showed that when analyzing gene expression data, permutation importance overestimates the importance of correlated predictor variables that are unassociated with the outcome. Nicodemus and Shugart (2007) [8] used simulated genetic data of a null model to show that the permutation importance measures are not biased. However, it is unknown how and to what extent LD between non-causal SNPs and true risk SNPs affects the ability of Random Forests (RF) to identify the true risk SNPs. Arguably, the correlation would lead to diminished variable importance for each risk SNP that has non-causal SNPs correlated with it. If this is the case, the LD among SNPs will affect our ability to identify which specific polymorphisms are responsible for increased disease risk, and may even hinder our ability to determine that any genetic factors influence the disease. If there were too many SNPs in LD, we might miss the genetic effect of the risk SNPs. In order not to miss indirect evidence, in some of the analyses we performed, we consider any of the SNPs in LD with the causal SNP as equivalent to risk SNPs. There are multiple ways to accommodate SNPs in LD within a RF analysis. Here, we explore two alternative approaches to address this problem. One way to deal with the problem presented by SNPs in LD is to build each tree in an RF only using SNPs in LE, and then to create a new importance measure for variables to account for the revised tree-building method. A second approach is to use haplotypes instead of SNPs to build a RF. We first present a simulation study exploring the power of the revised tree building method and compare it with the original RF method when the risk SNPs are in LD with non-causal SNPs. We then present a simulation study of the haplotype-based method and compare it with the SNP-based method when there is LD between risk SNPs. Finally, we present an example based on a genome wide study of association of SNPs with Alzheimer disease.

## Results

We simulated two scenarios under complex diseases models: (1) the risk SNPs are in LD with non-causal SNPs, and (2) the risk haplotypes are "responsible" for the risk instead of individual risk SNPs. For the first scenario, to demonstrate the effect of LD on SNPs using the original RF and the revised RF, we simulated a wide spectrum of complex genetics models (Table 1). For the second scenario, we simulated an H4M4 model, which includes a set of 16 risk haplotypes (rHAPs) in linkage equilibrium, interacting in independent quartets to increase disease risk.

**Table 1:** Genetic models for simulating risk SNPs for case control data.

		**Allele**	**Marginal GRR**		**Penetrance Factors**			**K**	**λ**_**s**_	**# Kept**
**Model**	**Number**	**Frequency**	**Het**	**Hom**	**0**	**1**	**2**			

H1M1	1	0.03	∞	∞	0	0.9	0.9	0.05	9	K1S1

H2M3	6	0.125	5.22	5.22	0	0.9	0.9	0.02	9	K3S3

H3M3	9	0.106	3.47	3.47	0	0.9	0.9	0.02	9	K3S3

H3M4	12	0.176	2.54	2.54	0	0.9	0.9	0.02	6	K4S4

H9M2	18	0.031	3.02	3.02	0	0.9	0.9	0.03	9	K2S2

H4M4	16	0.282	1.63	1.79	1.20E-08	0.79	1	0.10	2	K4S4/K4S2

H8M4	32	0.214	1.34	1.4	2.80E-03	0.86	1	0.10	2	K4S4

### A Random Forest in which individual trees are built only with SNPs in linkage equilibrium

**Figure 1:**
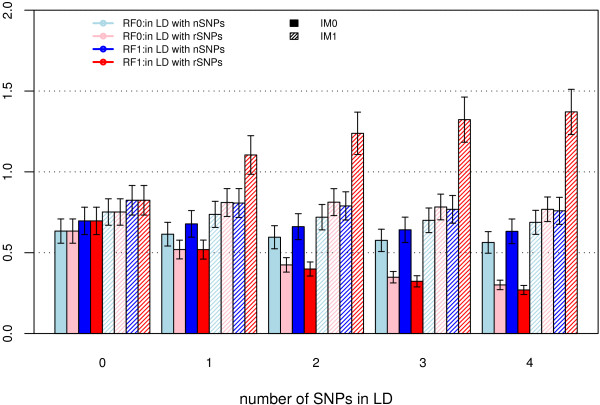
**Mean IM of rSNP when there are SNPs in LD with rSNPs**. The data were simulated using model H4M4 with K4S4 analysis design. Four importance measures are illustrated in the figure using original random forest (RF0) and revised random forest (RF1) respectively. Solid color stands for original importance measure (IM0), i.e. the importance measures were averaged over all trees in a Forest. Shaded color stands for the revised importance measure (IM1), i.e. the importance measures were averaged over trees containing the variable.

We compared the performance of the original RF and our proposed revised RF, using the original IM defined by Breiman and Culter [1,9] and revised IM. Under all disease models (Table 1), the original IM for functional risk SNPs decreased as the number of SNPs in LD with risk SNPs increased (Figure 1). The differences between the original IM combined with the original RF and the original IM combined with the revised RF are very small for fixed numbers of SNPs in LD with risk SNPs, indicating that using the original IM combined with either the original RF or the revised RF produces similar results when there are non-causal SNPs in LD with risk SNPs. The revised IM combined with original RF does not show a consistent pattern across the different genetic models as the number of SNPs in LD with risk SNPs increases. Under all genetic models, the revised IM combined with the original RF produces more stable IMs as the number of SNPs in LD with risk SNPs increases than does the revised IM combined with the revised RF. The revised IM combined with the revised RF tends to produce inflated IM as the number of SNPs in LD with risk SNPs increases. Thus, the revised IM combined with the original RF has the most stable performance when there is LD (Figure 1).

**Figure 2:**
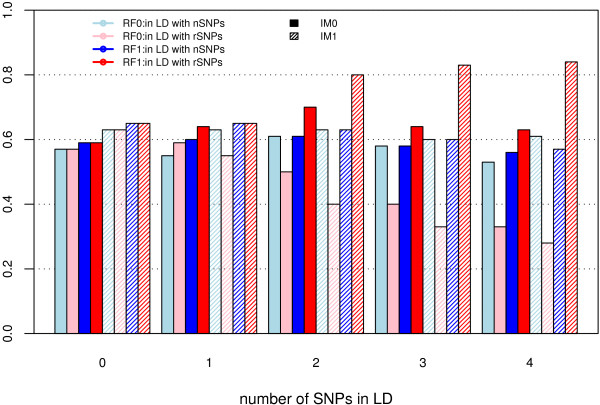
**Proportion of replicates where IM(rSNP and LD.rSNP) > maxIM(nSNP)**. The data were simulated using model H4M4 with K4S4 analysis design. The proportion of replicates where IMs of all rSNPs and LD.rSNPs – IM(rSNP and LD.rSNP) – exceeded the maximum IM of the noise SNPs – maxIM(nSNP).

Proportion of replicates where all rSNPs and LD.rSNPs have higher IMs than noise SNPs showed a different trend. The revised RF combined with the original IM had the most stable performance as the number of LD.rSNPs increased (Figure 2). This result suggests rSNPs and LD.rSNPs exist in the same tree, and thus hurt the importance measure using original RF.

**Figure 3:**
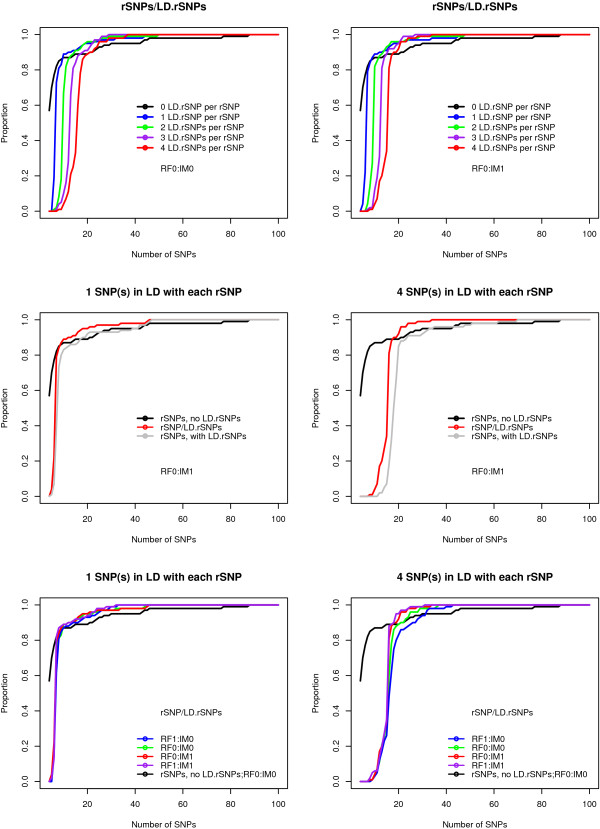
**Proportion of replicates for which all rSNPs and/or LD.rSNPs are among the top-ranking X SNPs**. The data were simulated using model H4M4 with K4S4 analysis design. (A) Top panels: the proportion of replicates for which each rSNP or one of its corresponding LD.rSNPs is among the top X SNPs ("rSNPs/LD.rSNPs"). Left panel: RF0:IM0; right panel: RF0:IM1. (B) Middle panels: using RF0:IM1, we compare the proportion of replicates for which each rSNP or at least one corresponding LD.rSNP was among the top-ranking X SNPs, the proportion considering only rSNPs ("rSNPs with LD.rSNPs"), and the proportion where there are no rSNPs in the dataset ("rSNPs, no LD.rSNPs"), with example of 1 SNP and 4 SNPs in LD with each rSNP. (C) Bottom panels: we compare the four combinations of original and revised RF and original and revised IM, with examples for 1 and 4 LD.rSNPs.

For all models and all four combinations of original and revised RF and IM, the proportion of replicates for which each rSNP or at least one of its corresponding LD.rSNPs is among the top-ranking X SNPs is smaller when there is one or more LD.rSNP than the proportion when there are no LD.rSNPs when X is small (8~20) (Figure 3). However, as X increases to 20 or greater, the proportion for data sets with at least one LD.rSNP becomes larger than the proportion for data sets with no LD.rSNPs. We show this trend using the original IM and the revised IM with the original RF algorithm in Figure 3 (top panels). When we compare datasets with different number of LD.rSNPs, the proportion of replicates for which each rSNP or at least one corresponding LD.rSNP was among the top-ranking X SNPs was higher than the proportion considering only rSNPs in some situations. We see this in Figure 3 (middle panels) when X ≥ 8 and there is 1 LD.rSNP), and for X ≥ 20 when there are 4 LD.rSNPs. Thus, when considering the identification of LD.rSNPs equal to the identification of rSNPs, including the LD.rSNPs in the analysis is more powerful than using rSNPs alone under some conditions. In Figure 3, bottom panels, we compare the four combinations of original and revised RF and original and revised IM for 1 and 4 LD.rSNPs. For a fixed number of LD.rSNPs, the original RF had better performance than the revised RF, and the original and revised IM had nearly identical performance within each RF method.

### Using haplotypes instead of SNPs as predictor variables

Another way of dealing with LD is to build an RF using haplotypes instead of SNPs, but not change the implementation of the original RF.

**Figure 4:**
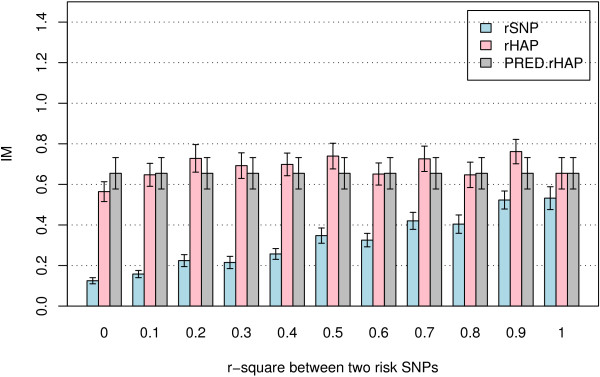
**Mean of IM(rSNP), IM(rHAP) and IM(PRED.rHAP)**. The data were simulated using model H4M4 with K4S4N100 analysis design. The importance measures are the original importance measures (IM0) combined with the original RF.

**Figure 5:**
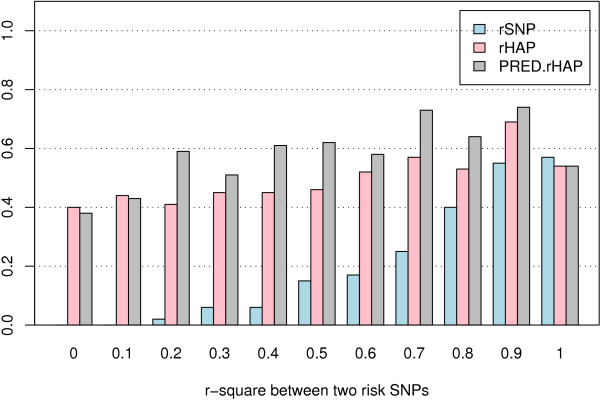
**Proportion of replicates where IM (rSNP) or IM(rHAP) or IM(PRED.rHAP) > maxIM(nSNP)**. The data were simulated using model H4M4 with K4S4 analysis design, and analyzed using the original RF. The proportion of replicates where the IMs of all rSNPs, or all risk haplotypes (rHAP), or all predicted haplotypes (PRED.rHAP) all exceeded the maximum IM of the noise SNPs – maxIM(nSNP).

**Figure 6:**
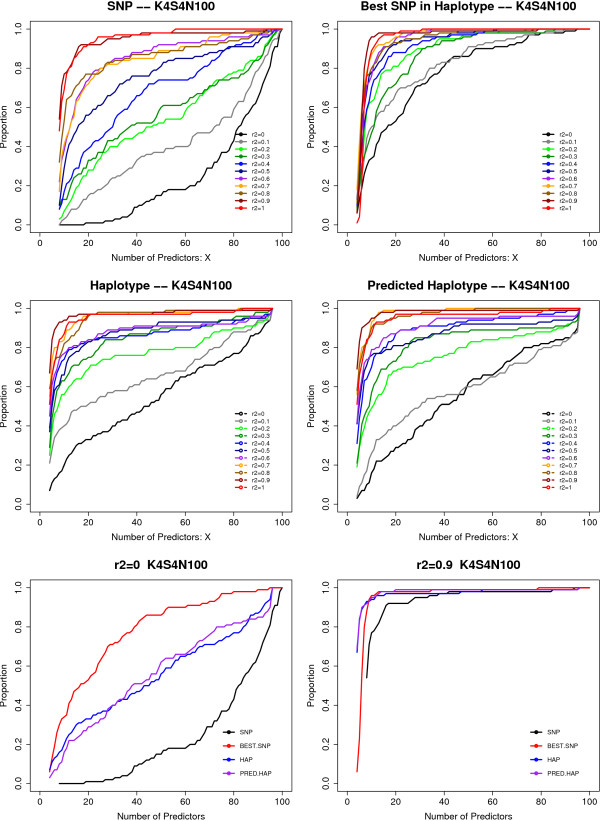
**Proportion of replicates for which all rSNPs or best rSNP in haplotype, and HAPs are among the top-ranking X SNP/HAPs**. (A) Top panels. Individual SNPs are used in the RF as the predictors. Left: The proportion of replicates for which both SNPs making up the haplotype are among the top X predictors. Right: The proportion of replicates where at least one of the two SNPs in the risk haplotype are among the top X predictors. (B) Middle panels: Left: true haplotypes are used in the RF as the predictors. The proportion of replicates where the true haplotype is among the top X predictors. Right: the haplotype phases are not known, and are resolved statistically. The predicted haplotypes are used in the RF as the predictors. (C) Bottom panels (left, right) show the four analyses for haplotypes consisting of two SNPs with r^2 ^= 0 and r^2 ^= 0.9.

Under the H4M4 model described in Table 1 with the K4S4N100 design, the IM for risk SNPs increased with increasing LD between the risk SNPs. The mean IM for risk haplotypes and predicted risk haplotypes was relatively stable as the LD between the risk SNPs increased. The results displayed in Figure 4 show that in general, the mean IM of the risk haplotype was higher than that of the predicted risk haplotype, which was higher than the mean IM of the risk SNPs that make up the haplotype used as independent predictors. The difference in IM among the three analysis options decreased as the strength of the LD between the risk SNPs in the risk haplotype increased (Figure 4). The proportion of replicates for which the IMs of all of the risk SNPs (for the SNP analysis) or risk haplotypes (for the haplotype methods) exceeded the maximum IM of the noise SNPs also increased as LD between the risk SNPs in the risk haplotype increased (Figure 5). The proportions of replicates for which all risk variables (risk SNPs or risk haplotype, depending on analysis method) were among the top-ranking X variables showed a similar trend (Figure 6). Since the two risk SNPs in the risk haplotype are correlated, identification of one should bring attention to the region. Therefore, we also examined the performance when the best risk SNP in the risk haplotype is considered. Importantly, the proportion of replicates for which at least one of the risk SNPs in each risk haplotype was among the top-ranking X variables was greater than the proportion of replicates where both risk SNPs in risk haplotype were among the top variables, and was also better than or close to the proportion for the risk haplotype or predicted haplotype as predictors. Due to the computational burden of calculating predicted haplotypes, the analyses using predicted haplotypes were performed only for the H4M4 model and K4S4N100 design, with 1000 trees for each random forest.

### Application in Alzheimer Disease GWAS data

We applied the random forest methods to a dataset from a recently published genome wide association study in Alzheimer Disease (AD) [10], which has been made publicly available (Translational Genomics Research Institute; TGEN, data downloaded from ). The widely acknowledged risk gene for AD is apolipoprotein E precursor APOE on chromosome 19. The TGEN dataset included 861 AD cases and 550 controls. The best proxy SNP for the APOE e 4 variant known to increase AD risk is rs4420638 [10]. Because no other SNPs in LD with the APOE e 4 variant were present in the dataset, we increased the genomic coverage in regions surrounding APOE by imputing the genotypes for 17,790 additional HapMap SNPs (Hapmap 2, CEU samples) within ~10 kb of rs4420638 using PLINK [11], resulting in a total set of 19,010 SNPs. Then, we randomly selected 250 cases and 250 controls, and selected 103 SNPs in the neighborhood of rs4420638, including three imputed SNPs (Table 2) in LD with rs4420638 (r^2 ^> 0.2). A second dataset was created excluding the three SNPs in LD with rs4420638 for comparison. We applied both original RF and the revised RF to the two datasets, calculating original IM and revised IM for each analysis.

**Table 2:** Correlation of three SNPs with rs4420638 in TGEN data.

**Chr**	**SNP**	**r^2^**
19	rs6859	0.23

19	rs6857	0.55

19	rs10119	0.68

19	rs4420638	1.00

For any combination of RF and IM, rs4420638 has relatively high importance and was ranked within the top 11 SNPs (Table 3). The original IM of rs4420638 combined with original RF decreases when there are SNPs in LD with it (0.28 vs. 0.1512 for dataset 2 and dataset 1). The revised IM combined with original RF performs a little better than original IM with original RF and original IM with revised RF performs a little worse. However, revised IM combined with revised RF yields the highest IM among the four, and is also the most similar to the value of original IM when we include no SNPs in LD. The trend for the four IMs ranks is similar. Then we examined whether additional SNPs are included in the top 11 ranked SNPs using the revised RF and/or revised IM applied in dataset 1 as compared to using original RF applied in dataset 2 (Table 4). The revised IM combined with original RF calculated from dataset 1 identified two additional SNPs, including one SNP (rs10119) in LD with rs4420638; revised IM combined with revised RF from dataset 1 identified three additional SNPs. Out of these 17 SNPs identified as top 11 ranked SNPs for each of the four combinations of RF and IM, 7 SNPs would have been missed using single SNP analysis approach (nominal p > 0.05 for both genotypic association and allelic association).

**Table 3:** Results of RF analysis on TGEN data: IM and rank of rs4420638.

	**Dataset 1: 103 SNPs**		**Dataset 2: 100 SNPs**	
	**IM**	**Rank**	**IM**	**Rank**

RF0:IM0	0.1512	9	0.28	3

RF0:IM1	0.1519	9	0.2803	6

RF1:IM0	0.1436	7	0.3123	4

RF1:IM1	0.3191	11	0.3123	10

**Table 4:** Results of RF analysis on TGEN data: Top 11 ranked SNPs, and single SNP association analysis.

**Dataset 1**							
		**RF0:IM0**	**RF0:IM1**	**RF1:IM0**	**RF1:IM1**	**GENO**	**ALLELIC**

**rs2927477**	50005555	+	-	-	-	0.4483	0.4122

rs4803759	50019299	+	+	+	+	0.0833	**0.0242**

rs4605275	50030333	+	+	+	+	0.0962	**0.0291**

**rs8104483**	50064194	+	+	+	+	0.6459	0.4007

**rs4803767**	50064799	+	+	+	+	0.6459	0.4007

rs10119	50098513	+	+	-	-	**0.0028**	**0.0055**

rs4420638	50114786	+	+	+	+	**2.651E-12**	**7.73E-12**

**rs5158**	50139018	+	+	+	-	0.3015	0.8121

**rs3760627**	50149020	-	-	-	-	0.6852	0.5458

**rs16979595**	50169221	-	-	-	-	0.9526	0.8689

**rs7257916**	50174724	-	-	-	-	0.6859	0.5471

rs10424046	50227876	+	+	+	+	**0.0106**	**0.0038**

rs1560725	50235627	+	+	+	+	0.0507	**0.0466**

rs11083758	50238901	-	-	+	+	**0.0474**	**0.0134**

rs3786507	50240095	+	+	+	+	**0.0374**	**0.0396**

rs2889490	50242247	-	-	-	+	**0.0252**	**0.0094**

rs10416445	50248502	-	-	+	+	**0.0187**	**0.0065**

**Dataset 2**							

		**RF0:IM0**	**RF0:IM1**	**RF1:IM0**	**RF1:IM1**	**GENO**	**ALLELIC**

**rs2927477**	50005555	+	-	-	-	0.4483	0.4122

rs4803759	50019299	+	+	+	+	0.0833	**0.0242**

rs4605275	50030333	+	+	+	+	0.0962	**0.0291**

**rs8104483**	50064194	+	+	+	+	0.6459	0.4007

**rs4803767**	50064799	+	+	+	+	0.6459	0.4007

rs10119	50098513	NA	NA	NA	NA	**0.0028**	**0.0055**

rs4420638	50114786	+	+	+	+	**2.651E-12**	**7.73E-12**

**rs5158**	50139018	+	+	+	-	0.3015	0.8121

**rs3760627**	50149020	-	+	-	-	0.6852	0.5458

**rs16979595**	50169221	+	-	-	-	0.9526	0.8689

**rs7257916**	50174724	-	+	-	-	0.6859	0.5471

rs10424046	50227876	+	+	+	+	**0.0106**	**0.0038**

rs1560725	50235627	+	+	+	+	0.0507	**0.0466**

rs11083758	50238901	-	-	+	+	**0.0474**	**0.0134**

rs3786507	50240095	+	+	+	+	**0.0374**	**0.0396**

rs2889490	50242247	-	-	-	+	**0.0252**	**0.0094**

rs10416445	50248502	-	-	+	+	**0.0187**	**0.0065**

## Discussion

We compared the performance of the original and the revised RF, combined with the original and revised IM when there are SNPs in LD with the functional risk SNPs under various genetic models, in terms of mean importance measure, the proportion of replicates where IMs of all risk SNPs and SNPs in LD with the risk SNP exceeded the maximum IM of noise SNPs, and the proportion of replicates for which all risk SNPs are among the top-ranking X SNPs. The simulations indicate that the stronger the genetic effect, the stronger the effect LD has on the RF performance. For most of the genetic models we simulated, the revised IM demonstrated better performance than the original IM when used with either the revised Random Forest method or the original Random Forest method. The revised IM with original RF showed the most stable performance overall. However, for the proportion of replicates where IMs of all risk SNPs and SNPs in LD with the risk SNP exceeded the maximum IM of noise SNPs, and revised IM with revised RF showed the best performance, and improved performance with increasing numbers of SNPs in LD with risk SNPs. Although we do not know a priori which SNPs are risk SNPs, we have found that SNPs in LD with noise SNPs have little effect on the performance (data not shown), suggesting the advantages of including all SNPs in analyses.

In terms of assigning higher ranks to all risk SNPs than to noise SNPs, the simulations showed that the performance of the RF method decreased when there are SNPs in LD with the risk SNPs. However, if risk SNPs and SNPs in LD with the risk SNPs are considered equally valid "hits" when trying to identify an association, inclusion of these correlated SNPs increased the probability that all "hits" (risk SNPs or SNPs in LD with risk SNPs) were among the top-ranking X SNPs as compared to risk SNPs only. This finding suggests that the RF method may be a good alternative to other methods such as multivariable logistic regression to detect association when there is correlation among SNPs. We have shown that for some genetics models, the original RF had reasonably good performance when there were SNPs in LD with risk SNPs. A plausible reason lies in the nature of the tree building method of random forest, where if an important variable SNP1 is selected near the root, the variable SNP2 that is highly correlated with SNP1 will be very unlikely to be the "best" variable to split on if it is among the few randomly selected variables chosen for one of the child nodes. Thus, SNP2 will likely to be closer to a leaf node if selected at all. Similarly, if SNP2 is selected first, SNP1 will be near a leaf node. We used an example for the comparison of the IMs using the original RF and the revised RF. In the original RF, in some trees, SNP1 is near the root and SNP2 is close to the leaf; in some trees, SNP2 is near the root and SNP1 is close to the leaf. The permutation of the SNP1 value will greatly increase the prediction error of that tree when it is near the root; however, the permutation of the SNP1 value will not increase the prediction error of the tree when it is near the leaf node, largely because SNP2 can act as surrogate for SNP1. However, it might still increase the prediction error slightly. Therefore, SNP1 in this tree might contribute positively to the average IM over all trees. In the procedure of the revised RF method, the first selection of SNP2 excludes the possibility of the selection of SNP1 in the same tree and visa versa. Therefore, there is no positive contribution of SNP1 to the average original IM from this tree as in the original tree. Overall, with a forest of trees, the average original IM of SNP1 over all trees might decrease due to this intervention. However, the revised IM is averaged over only trees that contain this variable; therefore the final IM will not be affected in the same manner. Our reasoning is reconfirmed by an interaction test of the original random forest method [9]. Variables A and B are said to interact if a split on one variable, say A, in a tree makes a split on B either systematically less probable or more probable. The interaction test on the data with SNPs in LD produces a large value for the interaction test between the SNPs in LD, implying a split on one SNP inhibits a split on the other and vise versa. The stronger the genetic effect of this SNP, the less likely SNPs in LD will be in the same tree. In fact, the number of trees that contain the variable when there is a correlated variable is smaller than the number of trees that contain the variable when there is no correlated variable, i.e. the two correlated variables compete for trees. This further supports our reasoning above.

In summary, under genetic models with strong marginal effects, the original IM is sensitive to the number of SNPs in LD with risk SNPs; however, it is relatively robust to the problem of correlation among SNPs under genetic models with weak genetic effects. The revised IM combined with the original RF is relatively robust to LD among SNPs; the revised IM in the revised RF can be inflated. Therefore the combination of revised IM and original RF is a better choice when the genetic model and the number of SNPs in LD with risk SNPs are unknown.

Under the scenario where risk haplotypes are responsible for the risk instead of individual risk SNPs, we simulated noise SNPs independent of disease status. The findings of these analyses suggest that when a haplotype is responsible for increased disease risk, using the haplo-genotypes as predictors is in general more powerful than using the genotypes of the individual SNPs comprising the haplo-genotype as predictors. The differences decreased with increasing LD between the risk SNPs in the risk haplotypes. The performance of predicted haplo-genotypes as input was between that of the risk SNPs and that of the risk haplotypes, but was more similar to that of the risk haplotypes. The difference in performance between the predicted haplo-genotype and true haplo-genotype analyses was greatest when the risk SNPs in risk haplotype were not in LD.

The decrease of performance of the risk SNPs comprising the risk haplotype as predictors as compared to using the risk haplotype greatly depended on the level of LD between the SNPs in the risk haplotype. We observed the greatest decrease in performance for individual SNPs compared to haplotypes when there was no LD (r^2 ^= 0) between the two risk SNPs making up the risk haplotype. This trend was expected given the nature of the correlation. In practice, however, when there is low LD, we usually would not infer haplotypes. In other words, if we only infer haplotypes when there is substantial LD, the gain in performance compared to individual SNPs is limited. Moreover, in the context of thousands (*n*) of SNPs, the computation time required for resolving haplotypes of all pairs of SNPs is O(n*n), and that for resolving haplotypes of *m *SNPs increases exponentially (*O*(*n*^*m*^)) and probably outweighs the benefit of using haplotypes instead of SNPs as predictor variables. However, in the same context, this computational burden is a problem for other analysis methods as well, and not a particular disadvantage of the RF method. When there was strong LD between risk SNPs comprising an risk haplotype (r^2 ^> 0.8), the decrease in performance for using individual SNPs instead of haplotypes was trivial; using risk SNPs as the predictors performed reasonably well compared to using the true risk haplo-genotypes. This is because the 2-locus genotype is an increasingly better surrogate for the haplo-genotype as the LD increases. This is understandable because the haplotypes carry information from 2 SNPs, when there is no LD between the 2 SNPs, each single SNP carries half of the total information, i.e. 50% of the information of the haplotype. When there is complete LD between 2 SNPs, each SNP carries the same information as the total information of the haplotype, i.e. 100% of the information of the haplotype.

We applied the random forest methods in a recently published GWAS data from TGEN. Using random forest methods, we have successfully identified the known AD risk gene APOE, and also identified new candidate loci that are independent of APOE e 4 variant, which would have been missed using a single SNP approach.

Our results suggest that the RF method provides robust performance in the two scenarios discussed above without filtering the SNPs to remove those in LD or preprocessing to create haplotypes, making it a viable tool for use in context of thousands of SNPs. Genome wide association studies (GWAS) include several hundred thousand or more SNPs; the version of RF that we use does not handle GWAS data because of the memory requirements. We performed our analyses on a Linux cluster – an IBM e1350 solution configured with a head node, a storage node w/scsi RAID storage enclosure, and 134 dual Intel Xeon 2.8 GHz cpus blade servers. 110 nodes have 1 GB RAM and 24 nodes have 2 GB RAM. With 1500 individuals and 10 K SNPs, we are only able to grow 17,500 trees in the forest using the Fortran version; the R version can grow about 15000 trees. With a smaller sample size (500), more trees can be grown, and more variables can be handled. However, analyzing 500 K SNPs at once will be challenging. At the 2008 International Genetic Epidemiology Society meeting, a new RF algorithm ("random jungle" ) was proposed that may be appropriate for full genome wide SNP analysis with 500 k SNPs or more. Often in practice, only the top markers (i.e., several hundred or thousands) of SNPs are used for further data-mining. It is likely that many SNPs are in LD, and it is possible that haplotypes are either true risk factors, or better surrogates for a non-genotyped functional SNP than individual SNPs that are genotyped. While many fewer SNPs are considered in candidate gene studies, the extent of LD among SNPs in these studies is likely to be high as well.

Another practical issue relating to the use of random forests to identify important SNPs is determining an appropriate threshold for the IM score without knowing the number of SNPs involved in the disease. There are several ways to make the decision, depending on the goal of the analysis. If the goal is merely to rank the SNPs and select the top variables, this can be achieved using various methods, for example, by the IM distribution curve [12] or by the iterative random forest procedure [13,14]. If the goal is to know whether the IM is higher than expected by chance, this can be achieved by evaluating the significance level of IM by randomly permuting sample outcome phenotypes. In any case, because the IM measure is not stable, one should build multiple random forests using different seeds to determine how much IM of the variables vary.

## Methods

### A Random Forest in which individual trees are built only with SNPs in linkage equilibrium

#### Revised RF tree building algorithm

When risk SNPs are in LD with non-causal SNPs, it can be predicted that the correlation would lead to diminished variable importance for each correlated risk SNP. Assuming that the risk SNP and the non-causal SNP in LD are in the same tree of the random forest, when the genotypes of the risk SNP (a node in a tree) are permuted randomly among samples, the non-causal SNP (another node in the same tree) will serve as its surrogate and the tree will still accurately predict case status. Over all trees in a forest, prediction error is not likely to increase much if the risk SNP is permuted because the surrogate SNP will take its place in predicting the phenotype. Hence, the importance measure (IM) of the SNP will not be high. If we build a forest such that within any tree, only one of the two SNPs in LD are present, the SNPs cannot act as surrogates for each other and the IM of the risk SNP should not be diminished. We implemented this strategy by building each tree in the RF only with SNPs in linkage equilibrium using Fortran source code of random forests (version 5.1) by Breiman and Cutler [15]. The <<buildtree>> function was revised by keeping track of variables selected at each node, and excluding from the selection pool of variables for the remaining nodes in the tree all SNPs with a pairwise genotypic correlation (r^2^) greater than a pre-specified value R with any SNP already used in the tree. Thus, variables with r^2 ^> R never appear in the same tree. We call this strategy "RF1".

#### Modified Variable Importance

A random forest uses all available covariates to predict response. Here, we use measures of variable importance to determine which covariates (SNPs, in our case) or sets of covariates are important in the prediction. Two importance measures are available in the Breiman and Cutler [15] RF implementation: permutation importance and Gini importance. The Gini importance is based on the Gini impurity criterion. However, it has been shown to be biased in a number of conditions [8,16]. Therefore, we use the permutation importance defined by Breiman [1]. This importance measure quantifies the importance of a predictor variable by disrupting the dependence between the variable and the response and measuring the change in the tree votes compared to the original observations. In practice, this is achieved by permuting the variable values among all observations in the out-of-bag sample of each tree. If the variable is predictive of the response, it will be present in a large proportion of trees and be near the root of those trees. Observations with a changed variable value may be directed to the wrong side of the tree, leading to vote changes from the right to the wrong class. Conversely, if the variable is not related to the response, it will be present in few trees and, when present, it will be near a terminal node, so that few tree votes will be changed. In Random Forests (version 5.1) [15] Breiman and Cutler define the importance index as follows. For individual i, let **X**_i _represent the vector of predictor variable values, y_i _its true class, V_j_(**X**_i_) the vote of tree j and t_ij _an indicator taking value 1 when individual i is out-of-bag for tree j and 0 otherwise. Let **X**^**(A,j) **^= (**X**_**1**_^**(A,j)**^,..., **X**_**N**_^**(A,j)**^) represent the vector of predictor variables with the value of variable **A **randomly permuted among the out-of-bag observations for tree j, and **X**^**(A) **^the collection of **X**^**(A,j) **^for all trees where N is the total number of individuals in the sample. In the special case of two classes, letting 1(C) denote the indicator function taking value 1 when the condition C is true and 0 otherwise, the importance index averages over the trees of the forest, and is defined as:

(1) $$I_{T}(A)=\frac{2}{T}\sum_{j=1}^{T}\frac{1}{N_{j}}\sum_{i=1}^{N}[1(V_{j}(X_{i})=y_{i})-1(V_{j}(X_{i}^{\left(A,j\right)})=y_{i})]t_{ij}$$

where *N*_*j *_represents the number of out-of-bag individuals for tree *j *and *T *is the total number of trees.

If all variables are independent, each variable will only be present in a subset of all the trees. The proportion of trees containing each variable is approximately the same for all variables. Assuming the proportion is P, it is easy to see in formula (1) that replacing *T *with *PT *generates the same ranking list of all SNPs. However, for our proposed new strategy RF1, the SNPs in LD cannot be in the same tree. This reduces the number of trees in which any SNP in LD with another SNP is present to less than *PT*, while SNPs independent of all other SNPs are still expected to appear in proportion *P *of all trees. Based on this observation, we modified the importance measure for the RF1 tree building strategy by replacing *T *with *T*_*v*_, the total number of trees in which the variable *v *appears. Formula (1) then becomes:

(2) IT'(A)=2Tv∑j=1Tv1Nj∑i=1N[1(Vj(Xi)=yi)−1(Vj(Xi(A,j))=yi)]tij

For each variable, we provide in the output the number of trees in which the variable appeared together with the original IM and revised IM.

### Simulation of SNP data

Association studies for complex phenotypes consider genotypes for hundreds or thousands of SNPs, either derived from genome wide or candidate gene association studies. In order to understand the effect of LD on the performance of random forest methods, we simulated a multiplicative heterogeneity model with various numbers of risk SNPs, SNPs not associated with the disease, and SNPs in LD with risk SNPs or non-risk SNPs. The total number of independent SNPs (both risk SNPs and non-risk SNPs) in our simulation is 100. The simulation is based on the models described by Lunetta *et al*. [2]. This section will briefly describe the methods. Additional details are provided in the original paper.

#### Genetic Models

Several complex disease models were simulated with sibling recurrence risk ratio for the disease (λ_s_) ranging from 2 to 9 and population disease prevalence (*K*_*p*_) ranging from 0.02 to 0.10. Genetic heterogeneity and multiplicative interaction as defined by Risch [17] were incorporated into the genetic models. The "multiplicative" in Risch's definition of "multiplicative model" is different from the one from standard statistical modeling, where, in the absence of a genetic interaction the risk of a double-mutant is expected to be the multiplicative product of the individual risks of the corresponding single mutants. Risch defines multilocus multiplicative and heterogeneity models in terms of penetrance factors. We use a two-locus model consisting of two allele SNPs as an example to illustrate these models. The genotypes at the first and second SNP are denoted by A_i_, i = *0, 1, 2*, and B_j_, j = *0, 1, 2*, respectively, where the subscript denotes the number of risk alleles. For a multiplicative model, we define p = (*p*_0_, *p*_1_, *p*_2_) and q = (*q*_0_, *q*_1_, *q*_2_) such that the penetrance of genotype A_i_B_j _is *w*_*ij *_= *p*_*i *_*q*_*j*_, then the p's and q's are referred to as "penetrance factors" for SNPs A and B respectively. For an additive model, the penetrance for genotype A_i_B_j _is *w*_*ij *_= *p*_*i*_+*q*_*j*_, and for a heterogeneity model, *w*_*ij *_= *1 *- (*1 *- *p*_*i*_) (*1 *- *q*_*j*_). The heterogeneity model is thought to be more realistic than the additive model because penetrances are always smaller than or equal to one. For our simulations, combined heterogeneity and multiplicative models were denoted using the shorthand introduced by Lunetta *et al*.[2] and summarized here:

HhMm:

H – number of heterogeneous systems

M – number of multiplicatively interacting SNPs within each system

For example, 16 SNPs are responsible for models H4M4 (Table 1).

To simplify matters, for all of our simulation models we assume that the penetrance factors for 0, 1, and 2 risk alleles are the same at each SNP: q = (q_0_, q_1_, q_2_).

Define a multi-locus genotype G = {g_11_, g_12_,..., g_HM_} where the first subscript h = 1,..., H denotes the heterogeneous system and the second subscript m = 1,..., M denotes the number of multiplicatively acting loci in each system. Each g_hm _is equal to 0, 1, or 2, denoting the number of risk alleles the individual caries at locus (h,m). Then the penetrance for genotype G is calculated as: $w_{G}=1-\prod_{h=1}^{H}\left[1-\prod_{m=1}^{M}q_{g_{hm}}\right]$

For the purpose of testing the effect of LD in the revised method (RF1), the H1M1, H2M3, H3M3, H3M4, H9M2, H4M4 and H8M4 models summarized in Table 1 were simulated to cover a large spectrum of complex disease models. We use the H4M4 model as an example to demonstrate the simulation. In this H4M4 model, sets of 16 risk SNPs were simulated such that the four groups of four risk SNPs account for the same proportion of the genetic risk, the four risk SNPs in each group follow a multiplicative model to increase disease risk, and at the population level, each risk SNP contributes equally to λ_s _and *K*_*p*_. Thus, the simulated risk SNPs all have the same allele frequency and the same observed marginal effect in the population.

#### Simulation of 13 LD models

In association studies, it is likely that only a subset of all risk SNPs contributing to a trait is genotyped. Therefore, in our simulation, we included only a subset of the total number of risk SNPs in each dataset. Following Lunetta *et al*. [2], we denoted the analysis design using the shorthand "KkSs" where "k" is the number of risk SNPs genotyped in the study and "s" is the number of genotyped SNPs within each multiplicative system. For the genetic model H4M4, a K4S4 design means that out of the total of 4 × 4 = 16 risk SNPs that contribute to the trait, four are genotyped, and that all four risk SNPs come from within one multiplicative set, and the other three heterogeneous systems are not represented at all in the dataset. In addition to the risk SNPs, we simulated independent noise SNPs not in LD with the risk SNP. These independent SNPs (100 SNPs in total) can be considered similar to tagging SNPs in real data. Finally, we add SNPs in LD with the independent risk and noise SNPs to mimic real SNP data.

We simulated SNPs in LD with the risk SNPs (SNPs 1 to 4 in the data file), and SNPs in LD with noise SNPs. We used acronyms for the different classes of SNPs:

(1) **rSNP (risk SNP)**: a SNP with a functional effect on the phenotype.

(2) **LD.rSNP**: a SNP in LD with a risk SNP, but not having its own functional effect on the phenotype.

(3) **nSNP (noise SNP)**: a SNP with no independent effect on phenotype, and not in LD with any rSNP or other nSNP.

(4) **LD.nSNP**: a SNP with no effect on phenotype that is in LD with other nSNPs

We treat the identification of SNPs in categories (1) and (2) as equally good, since the LD.rSNP identifies the correct region of the genome as associated with the trait. SNPs in categories (3) and (4) are "noise" SNPs that do not contribute any information about the phenotype.

We simulated 13 LD models. We simulated all LD using r^2 ^= 1, i.e. SNPs in LD are perfect proxies for each other. If LD has any effect on the performance of RF for identifying risk SNPs, we will see the most obvious effect when there is complete LD, and less effect for incomplete LD. First, we simulated 92 nSNPs (SNPs 5–96) in linkage equilibrium with allele frequencies distributed equally across the range 0.01–0.99 and an additional 4 nSNPs (SNPs 97–100) with allele frequencies of (0.3, 0.4, 0.5, 0.6), which were used to simulate LD.nSNPs for other LD models. The dataset containing 4 rSNPs + (92+4) nSNPs = 100 independent SNPs is called model n0, meaning "no LD among SNPs". For LD model n1, we simulated 1 LD.nSNP with each of the four nSNPs (SNPs 97–100). So in LD model n1, there are 4 rSNPs + (92+4) nSNPs + 4 LD.nSNPs = 104 SNPs. For LD model r1, we simulated one LD.rSNP for each rSNP. Thus, there are 4 rSNPs + (92+4) nSNPs + 4 LD.rSNPs = 104 SNPs. Similarly, we created the other 10 models as in Table 5. We generated 100 replicate data sets for each of the 13 LD models, each with 250 cases and 250 controls. For other genetic models, we simulated 100 SNPs for LD model n0, and simulated the other 12 LD models incrementally.

**Table 5:** Simulations of 13 LD Models for H4M4 model.

**Number of SNPs of each class**						
**LD models**	**rSNP**	**nSNP**	**nSNP**	**LD.rSNP per rSNP**	**LD.nSNP per SNP97:100**	**Total**

	SNP1:4	SNP5:96	SNP97:100			

**n0**	4	92	4	0	0	100

**n1**	4	92	4	0	1	104

**n2**	4	92	4	0	2	108

**n3**	4	92	4	0	3	112

**n4**	4	92	4	0	4	116

**r1**	4	92	4	1	0	104

**r2**	4	92	4	2	0	108

**r3**	4	92	4	3	0	112

**r4**	4	92	4	4	0	116

**r1n1**	4	92	4	1	1	108

**r2n2**	4	92	4	2	2	116

**r3n3**	4	92	4	3	3	124

**r4n4**	4	92	4	4	4	132

#### Simulation Analysis for SNPs

All analyses were performed on each of 100 replicate data sets of 250 cases and 250 controls for each of the 13 LD models. We treated the SNP genotypes as categorical predictors. In the original RF, a random selection of the potential predictors was used to determine the best split at each node. In the revised RF, each tree in a forest was grown on a subset of the predictors that are not in LD. So, within a tree, SNPs that are in LD would not be competing with each other. As a consequence, if two highly correlated SNPs in LD are both near the roots of different trees, they should both produce high importance measure (IM). In other words, the evidence for association for either of them to the outcome is kept. Taking both prediction error and the variable importance measures into consideration, we used RF tuning parameters *ntree *(number of trees to grow in a random forest) of 5000 and *mtry *(a random subset of all the predictors chosen to determine the best split at each node in a single tree) of 35 for all the analyses in this paper. We tested a few *mtry *values, starting from the square root of the number of variables, and found that it does not affect the importance measure much, as indicated by Breiman [1], Lunetta *et al*. [2], and Bureau *et al*. [18].

We examined how the importance measures (IM) at the risk SNPs (IM(rSNP)) behave as the number of LD.rSNPs increases, and/or as the number of LD.nSNPs increases. The following statistics were calculated over 100 replicates of a model. The noise SNPs refer to nSNPs and LD.nSNPs.

(1) Mean of IM(rSNP) for all 13 LD models (Table 5);

(2) Proportion of replicates where IMs of all rSNPs and LD.rSNPs exceeded the maximum IM of the noise SNPs. It is the proportion of replicates where all top 4 (8, 12, 16, 20) SNPs are rSNPs when there are 0 (1, 2, 3, 4) LD.rSNP(s) for each rSNP.

(3) Proportion of replicates for which all rSNPs and/or LD.rSNPs are among the top-ranking X SNPs. Two statistics are calculated. The first is the proportion of replicates for which all rSNPs are among the top-ranking X SNPs; the second is the proportion of replicates for which each rSNP or one of its corresponding LD.rSNPs is among the top X SNPs.

### Simulation of Haplotype data

We simulated a scenario where risk haplotypes (rHAPs) are "responsible" for the risk instead of individual rSNPs. Noise SNPs (nSNPs) were simulated, independent of disease status, with allele frequencies distributed equally across the range 0.01–0.99. For example, if 99 nSNPs were simulated, the allele frequencies of these 99 nSNPs are 0.01, 0.02,..., 0.98, 0.99. For simplicity, a 2-SNP haplotype was simulated as the risk haplotype (rHAP). We simulated the H4M4 model, where in each heterogeneous system there are 4 interacting risk haplotypes. For the H4M4 model described in Table 1, the allele frequency for the risk SNP is 0.282 and we would like to set the haplotype frequency to be similar. However, in order to avoid unrealistic haplotype structure, for this model, we selected haplotype frequencies of the four possible haplotypes using empirical haplotype frequencies [T-C (0.570), **G-T(0.287)**, T-T (0.113), G-C (0.030); r^2 ^= 0.447, D' = 0.831] of two SNPs (rs1503415, rs10790447) in SORL1 on chr11 of Hapmap CEU dataset. The haplotype frequency of G-T is observed to be 0.287, similar to the 0.282 minor allele frequency in the H4M4 model described earlier. In order to evaluate the effect of LD between SNPs on the RF performance, we created 11 LD models by fixing the haplotype frequency of G-T at 0.287, and adjusting haplotype frequencies of the other three possible haplotypes to create a set of 11 possible combinations of haplotype frequencies, with LD (r^2^) between the two SNPs ranging from 0 to 1 with increment of 0.1. We denoted the analysis design using the shorthand KkSsNn. KkSs is used as described above for simulation of SNP data, but now we are referring to risk haplotypes (rHAPs), rather than risk SNPs. The value "n" is the total number of rHAPs and noise SNPs included in the dataset; for example, if n = 100 and k = 4, s = 4, then the number of noise SNPs = 100 - 4*2 = 92. We created datasets with K4S4N100 design and K4S2N100 design for all LD models. For the K4S4 design, within each set, 4 interacting rHAPs (i.e. 8 rSNPs) from one heterogeneous system were kept in the dataset; for K4S2N100 design, within each set, 4 rHAPs (i.e. 8 SNPs), from two heterogeneous systems (2 interacting rHAPs from each heterogeneous system) were kept in the dataset. The 92 nSNPs were simulated in linkage equilibrium with allele frequencies distributed equally across the range 0.01–0.99.

#### Simulation Analysis for Haplotypes

We examined three ways of coding the data. The first was to consider the two SNPs making up the haplotype as variables of interest, and treat the SNP genotype (rSNP) as a categorical predictor, with a maximum of three categories if all three possible genotypes are observed in a dataset. The second was to consider the true haplotype of 2 SNPs comprising the risk haplotype as the variable of interest, and treat the haplotype pair or haplo-genotype (rHAP) as categorical input covariates, with a maximum of 10 categories since there are 10 possible haplotype pairs for two SNPs when four haplotypes are observed. The third was to treat the two SNP genotypes as phase unknown, and resolve the haplotypes using the software package haplo.stats[19]. From the haplotype resolution routines in haplo.stats [19], we obtained the probability of a haplotype *x *(*f*(*x*)). Next, we assigned each individual a haplotype pair (i.e. phased genotype) by randomly choosing a haplotype pair according to the probability distribution *f*(*x*). Then we recoded the haplotype pair (haplo-genotype) for each individual as input to RF. We treated the predicted haplo-genotype (PRED.rHAP) as a categorical variable and applied RF. For each individual, we simulated 100 possible haplotype pairs with probability distribution *f*(*x*). For example, for each of the four risk haplotypes (2 SNPs), we obtained the individuals with the same ambiguous haplotype pairs and sampled 100 haplotype pairs based on posterior probability *f*(*x*). For 2-SNP haplotypes, only double heterozygotes have ambiguous haplotype (22/11 or 21/12). With this method, for each dataset, we created 100 simulation datasets coded using the predicted haplotype distribution (pred.hap.dat). Then, we ran RF on the 100 "pred.hap.dat"s, with PRED.rHAP for each individual used as the input variable. Importance measures for the PRED.rHAP were averaged over the 100 datasets, and variables were ranked by the averaged IM. All analyses were performed on 100 replicate data sets of 250 cases and 250 controls.

## Availability and requirements

Project name: Random Forests Linkage Disequilibrium project

Project home page: 

Operating system(s): The program was developed on Linux machine with g77 compiler. It was based on the original Random Forests code by Leo Breiman and Adele Cutler. It has not been tested on other platforms.

Programming language: FORTRAN77

License: GNU GPL.

## Authors' contributions

YAM and KLL conceived of and led the design of the study and drafted the manuscript. YAM implemented the revised random forest and revised importance measure, conducted all phases of simulation, and analysis. YY added critical insight to the development of the revised importance measure of random forest. LAC and LAF provided comments on draft manuscript, and read and approved the final manuscript.
